# The influence of chronotype on making music: circadian fluctuations in pianists' fine motor skills

**DOI:** 10.3389/fnhum.2013.00347

**Published:** 2013-07-09

**Authors:** Floris T. Van Vugt, Katharina Treutler, Eckart Altenmüller, Hans-Christian Jabusch

**Affiliations:** ^1^Institute of Music Physiology and Musicians' Medicine, University of Music, Drama, and MediaHanover, Germany; ^2^Lyon Neuroscience Research Center, CNRS-UMR 5292, INSERM U1028, University Lyon-1Lyon, France; ^3^Institute of Musicians' Medicine, University of Music Carl Maria von WeberDresden, Germany

**Keywords:** musician, piano, scale playing, practice, sensorimotor performance, chronobiology, chronotype, circadian fluctuation

## Abstract

Making music on a professional level requires a maximum of sensorimotor precision. Chronotype-dependent fluctuations of sensorimotor precision in the course of the day may prove a challenge for musicians because public performances or recordings are usually scheduled at fixed times of the day. We investigated pianists' sensorimotor timing precision in a scale playing task performed in the morning and in the evening. Participants' chronotype was established through the Munich Chrono-Type Questionnaire, where mid-sleep time served as a marker for the individual chronotypes. Twenty-one piano students were included in the study. Timing precision was decomposed into consistent within-trial variability (irregularity) and residual, between-trial variability (instability). The timing patterns of late chronotype pianists were more stable in the evening than in the morning, whereas early chronotype pianists did not show a difference between the two recording timepoints. In sum, the present results indicate that even highly complex sensorimotor tasks such as music playing are affected by interactions between chronotype and the time of day. Thus, even long-term, massed practice of these expert musicians has not been able to wash out circadian fluctuations in performance.

## Introduction

Periodic processes within biological organisms occur at various timescales, such as seconds (e.g., heartbeat or respiration), days (e.g., the sleep cycle), weeks (e.g., the circaseptan rhythms), or months (e.g., menstruation cycle). Those processes that operate on a roughly 24-h cycle are governed by the circadian clock, which provides a temporal structure that modulates biological functions to match the daily cycle with the environment (Roenneberg and Merrow, 2003; Merrow et al., 2005). The circadian clock is entrained to the 24 h in a day by so-called zeitgebers such as the light-dark cycle (Roenneberg and Merrow, 2003) but also other non-oscillating environmental factors (Roenneberg et al., 2003a).

Circadian fluctuations are evident in various physiological functions of the human organism: clinical chemical parameters and endocrinological parameters such as concentration of hemoglobin, potassium, iron, adrenaline, noradrenaline, cortisol, and other hormones in blood and serum (Wisser and Breuer, 1981), body temperature (Aschoff, 1955) as well as cognitive functions such as reaction time to sensory cues (Kleitman et al., 1938) and memory tasks of various complexity (Van Eekelen and Kerkhof, 2003). In particular, circadian fluctuations also occur within the sensorimotor system. Circadian maxima and minima have, for example, been reported in grip strength (Atkinson et al., 1993), elbow flexion torque (Gauthier et al., 1996), back and leg strength (Coldwells et al., 1994), finger-tapping (Dosseville et al., 2002), tracking tasks (Van Eekelen and Kerkhof, 2003), and manual dexterity (Monk and Kupfer, 2000). However, to our knowledge, no studies exist that investigate variations in musical performance according to the circadian cycle.

Investigation of circadian rhythms has an important caveat: considerable inter-individual differences exist in the circadian clock as well as in its entrainment. These differences result in individual preferences in the timing of sleep and wake commonly referred to as chronotypes. Different chronotypes have been described such as “larks” (early sleepers) and “owls” (late sleepers) (Roenneberg et al., 2003a,b). Questionnaires have been developed to establish the individual chronotype either by assigning people to various categories such as morning, evening and intermediate type categories (Horne and Ostberg, 1976). Alternatively, researchers have calculated the midpoint between sleep onset and wake up, referred to as the mid-sleep timepoint, as the phase reference point for the sleep cycle (Benoit et al., 1981; Roenneberg et al., 2003b).

Indeed, these inter-individual differences in the circadian clock may explain differences in circadian fluctuations. This has been reported for cognitive performance in a range of memory tasks (Petros et al., 1990; May et al., 1993; Hasher et al., 1999; Intons-Peterson et al., 1999; West et al., 2002), for the alerting component of attention (Matchock and Mordkoff, 2009) as well as for the sensorimotor system, revealing chronotype-induced changes in maximum voluntary muscle contraction and excitability of the motor cortex (Tamm et al., 2009), furthermore in influence of bright light on physical performance (Kantermann et al., 2012). Activity patterns of the neural networks during the day have also been reported to be chronotype-dependent (Peres et al., 2011). As a consequence of the chronotype-dependent properties of circadian performance fluctuations, recent studies on circadian rhythms in the performance of complex tasks were based on study designs that controlled for the chronotype. For example, such chronotype-controlled circadian studies were used to identify dual-task costs (Jasper et al., 2010) or soccer-related performance (Reilly et al., 2007).

Performing music is a complex task executed on different times of the day. Performing music at a professional level is regarded as one of the most complex tasks in human life (Münte et al., 2002). Playing in public requires the highest possible level of performance independent of the time of the day. In matinee concerts, expert musicians have to play in the morning whereas in evening concerts, they have to perform at night.

To our knowledge, to date no systematic investigation exists into performance fluctuations across the daily cycle in musicians and their potential association with the chronotype. The present study investigates the performance quality in a demanding music-related sensorimotor task in professional pianists and its association with the time of the day and the chronotype. In order to objectively quantify performance quality, pianists were measured playing musical scales (Jabusch et al., 2004). Scales are never played perfectly evenly, therefore we divided timing deviations in systematic deviations (*irregularity*) and trial-to-trial variability (*instability*) (van Vugt et al., 2012). The temporal deviations that are consistent within trials (irregularity) were previously found to be inaudible and mostly determined by neuromuscular constraints (van Vugt et al., 2013). Therefore, we hypothesize that this part of the variability does not change across the daily cycle. However, instability reflects trial-to-trial variability away from these consistent deviations and is therefore a likely candidate for circadian fluctuations. We therefore hypothesized that early sleepers would be more stable, but not more regular, in the morning than in the evening. Similarly, late sleepers are expected to be more stable, but not more regular, in the evening than in the morning.

## Methods

### Participants

22 piano students (8 females) were recruited from the student pool at the University of Music, Drama and Media in Hanover. Participants were 22.5 (*SD* = 2.9) years old. All were right-handed according to the Edinburgh Handedness Inventory except for two left-handed participants (laterality quotient: *M* = 90.7, *SD* = 11.6 in right-handed participants; −81.8 and −100 for the two left-handed participants). Two participants were in the “piano performance” study path and one was studying in the so-called solo class (postgraduate studies); all others were in the “music education” study path which has lower admission standards. None of the participants reported any neurological condition. Participants were not selected using chronotype criteria. Rather than focusing on very early or very late sleepers (larks or owls), we decided to investigate circadian fluctuations in a representative sample of music students.

### Procedure

Participants came to the lab on two different days and were recorded playing scales. One measurement happened at 8 AM (henceforth referred to as AM recording) and the other at 8 PM (henceforth referred to as PM recording). Participants avoided to play piano prior to the measurement on both study days except a warm-up of five minutes immediately before the recording. The order of the AM and PM measurements was counterbalanced. Participants played on a MP 9000 MIDI stage piano (Kawai, Krefeld, Germany). The keyboard's digital music interface (MIDI out) signal was captured on a PC using a commercially available sequencer software (Musicator Win, version 2.12; Music Interactive Technology, Bergen, Norway).

Participants were requested to play two-octave C-major scales beginning with the C (131 Hz) one octave below the middle C and ending with the C (523 Hz) one octave higher than the middle C. Ascending and descending scales were interleaved. The instruction to the participants was to play as evenly as possible and in a legato style at mezzo-forte loudness. In order to maximally challenge the motor system, participants were required to play fast. This was achieved through presenting a metronome beat at 160 BPM and instructing the participants to play at 4 notes per metronome beat, resulting in 10.7 keystrokes per second. Participants performed roughly 15 scales with the right hand and with the left hand using the conventional fingering (123123412312345 and reverse, where the numbers indicate the fingers from the thumb, 1, to the little finger, 5).

### Questionnaires: chronotype and practice habits

Sleep habits were assessed applying the Munich Chrono-Type Questionnaire (MCTQ) (Roenneberg et al., 2003b), which participants filled out after whichever of the two recording sessions was last. This questionnaire is a validated tool to identify the chronotype based on self-reported individual sleep times, considering work and free days separately. The mid-point between sleep onset and waking up serves as sleep phase reference point. The sleep phase reference point is identified following an established protocol reported by Roenneberg et al. (2004): The so-called mid-sleep time-point is identified for the sleep in nights before work days and referred to as MSW. Additionally, the mid-sleep time point for nights before free days (MSF) is identified. To adjust the mid-sleep time point for the fact that individuals typically accumulate sleep debt on work days and compensate for this on free days, an adjusted mid-sleep (MSFsc) is calculated as follows. We first calculate the average daily sleep duration or need (ASD) as follows: ASD = (X × SDW + (7 − X) × SDF)/7 where X is the number of work days per week, SDF is sleep duration on free days and SDW is sleep duration on work days. Then the adjusted mid-sleep time point is calculated as follows: MSFsc = MSF − 0.5 × (SDF − ASD).

Additionally, a researcher-developed questionnaire focused on practice history and temporal practice habits at present. In analogy to mid-sleep, the individual “mid-practice time” (MPT) was identified. Participants reported their amounts of piano practice for each of eight 3-h time windows throughout the 24-h day. Time-windows were weighted according to these respective practice amounts. This enabled us to identify the MPT-point as the mid-point in time between onset and end of daily practice taking into account rests in between practice sessions.

### Scale playing analysis

First, we calculated the *unevenness* measure that has traditionally been computed to assess temporal precision in scale playing (Wagner, 1971; Jabusch et al., 2004). We proceeded to calculate the note onsets for each correct scale run and the standard deviation of the inter-onset intervals (in ms). We calculated the median of these for each combination of hand, playing direction (inward or outward; inward being defined as radial playing direction, outward as ulnar playing direction) and recording within each participant. As a secondary measure, we recorded the downward velocity of the keystrokes (in MIDI units). This parameter indirectly influences loudness: the faster the keystroke, the louder the sound. For each scale run, we calculated the SD of the keystroke velocities and then pooled these by taking the median for each pianist and condition.

**Figure 1:**
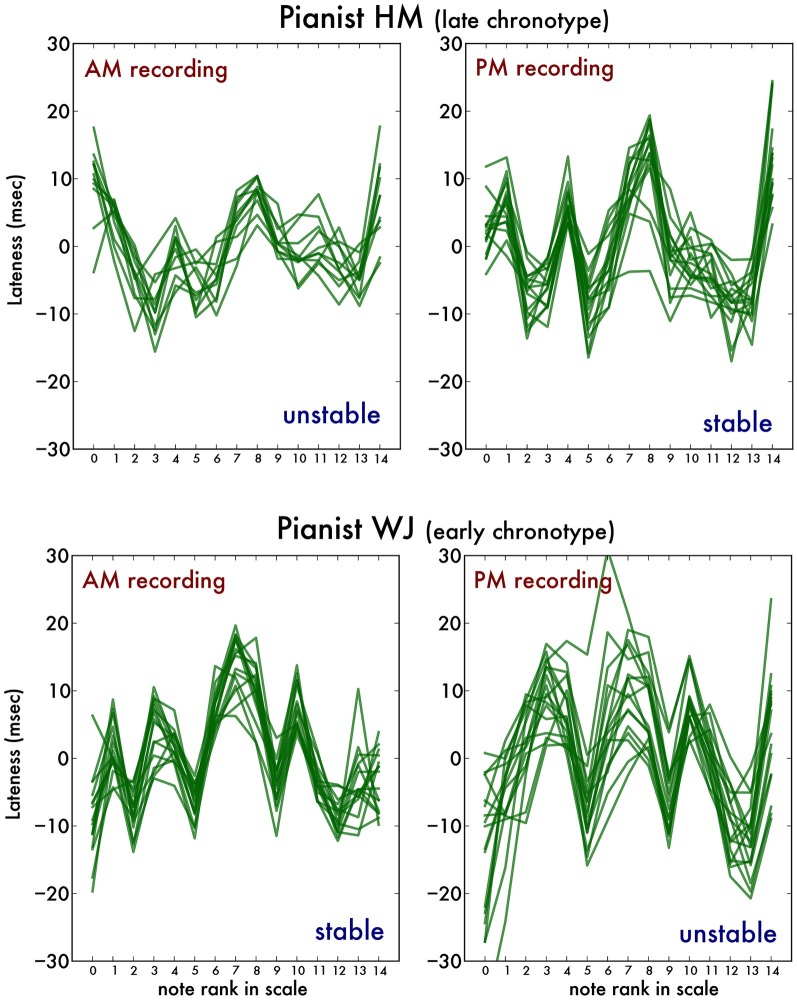
**Illustration of the timing analysis using two example pianists.** The green traces represent the lateness for each individual note in a dozen or so iterations of right hand ascending scale runs measured during each recording. Pianist HM (top plots) is of the late chronotype and pianist WJ (bottom plots) is of the early chronotype. The early chronotype pianist's playing is more stable during the morning (AM) than the evening recording (PM), whereas the opposite is true for the late chronotype pianist.

Secondly, we established note-by-note timing in scale playing according to a protocol published previously (van Vugt et al., 2012). First, we isolated correctly performed scale runs, discarding those containing errors or additional notes. We then converted the note values to their rank in the C major scale (i.e., C has rank 0, D has rank 1, E has rank 2, etc., up to C″ with rank 14) and performed a least-square straight line fit to this set of pairs of rank and timing. This allowed us to compute for each note the expected onset time (according to this fit) and then the deviation of the timing of the actually measured onset (in ms). We performed this fit for all scale runs and then pooled the results by hand (left or right), playing direction (inward or outward) and note, calculating the median lateness (*irregularity*; in ms) as well as inter-quartile range (*instability*; in ms) for that condition. Irregularity represents the amount of deviation away from regularity present within trials, capturing the fact that some notes are consistently late or consistently early within trials. Independent of this, the lateness of notes can vary between trials. Instability captures this by quantifying the variability in note timing across several trials. Figure 1 illustrates this procedure in two example pianists. It shows, firstly, that the pianists have highly individual timing traces (van Vugt et al., 2013). Secondly, in this example, the timing traces are more consistent (stable) for the early chronotype pianist's AM recording compared to the PM recording. The opposite is true for the late chronotype pianist. It is this observation that the following analysis captures. For more details on this procedure, the reader is invited to consult van Vugt et al. (2012). In sum, we segmented for each pianist the timing variability into variability due to deviations that are present across trials (irregularity) and variability between trials (instability).

Our ANOVAs were Type-II and we report the generalized effect size η^2^_G_ (Bakeman, 2005). Shapiro-Wilk normality test was used to verify normality of the data and Mauchly's test to detect sphericity violations, which were never significant for the data reported in this paper.

## Results

### Questionnaires: practice history, current practice habits and chronotype

One participant had to be eliminated due to technical reasons. The remaining 21 participants had started to play piano at the age of 5.5 (*SD* = 2.0) years and had played the instrument for a total of 17.0 (*SD* = 3.1) years, accumulating a total of 15.6 (*SD* = 7.5) thousand practice hours. The current daily practice duration was 4.0 (*SD* = 1.9) h. The median of the MPTs was 16.8 h local time, i.e., 4:48 PM (range, 13–20 h). The median number of work days per week was 7 (range, 4–7).

The MCTQ revealed an average sleep duration (total sleep duration on all work days and free days per week, divided by 7 days) of 7.7 (*SD* = 0.65) h. The corrected mid-sleep time-point on free days (MSFsc) was used as a proxy for the participant chronotype. The median of MSFsc was 5.0 h past midnight, local time (5:00 AM) (range, 4.1–6.2 h). A correlation was seen between participant's mid-sleep time point on work days (MSW) and MPT [Pearson *r*_(19)_ = 0.65, *p* = 0.001] indicating that pianists with an early sleep phase during workday nights practiced early during the day and vice versa. There was no correlation between MPT and mid-sleep time point on free days (MSF) [Pearson *r*_(19)_ = 0.26, *p* = 0.25], nor the corrected mid-sleep time on free days (MSFsc) [Pearson *r*_(19)_ = 0.33, *p* = 0.14].

For further analysis, a median-split procedure was carried out to classify participants as either an earlier chronotype (MSFsc < 5.0) or a later chronotype subgroup (MSFsc ≥ 5.0).

To check that our median split division in early and late chronotypes was legitimate, we verified that there were no differences in gender, age, age of commencement of piano training, number of years of piano training, nor in accumulated practice hours at the piano between the two groups (Table 1). The two groups clearly differed in sleeping behavior by definition of the group division.

**Table 1:** **Characterization of the late- and early chronotype groups**.

	**Early chronotype**	**Late chronotype**	**Statistical comparison**
Gender (female/male)	4/6	4/7	Fisher exact test *p* = 1.0
Age (years)	22.2 (3.65)	22.7 (2.28)	*t*_(14.8)_ = −0.39, *p* = 0.70
Handedness (Edinburgh laterality quotient %)	56.8 (78.6)	88.5 (11.53)	*t*_(9.4)_ = −1.26, *p* = 0.24
Age of commencement of piano training (years)	5.7 (1.87)	5.4 (2.25)	*t*_(18.9)_ = 0.37, *p* = 0.71
Amount of piano training (years)	16.5 (3.71)	17.3 (2.54)	*t*_(15.7)_ = −0.62, *p* = 0.55
Accumulated practice time (×1000 h)	16.3 (9.07)	15.0 (6.00)	*t*_(15.4)_ = 0.37, *p* = 0.71
Mid-sleep time before work days (MSW, hours after midnight)	4.4 (0.36)	4.9 (0.63)	*t*_(16.1)_ = −2.23, *p* = 0.04
Mid-sleep time before free days (MSF, hours after midnight)	5.0 (0.41)	5.6 (0.44)	*t*_(18.9)_ = −3.09, *p* = 0.006
Corrected mid-sleep time on free days (MSFsc, local time in hours after midnight)	4.6 (0.22)	5.3 (0.47)	*t*_(14.4)_ = −4.68, *p* = 0.0003
Mid-practice time (MPT, hours)	15.9 (2.31)	16.8 (1.35)	*t*_(14.2)_ = −1.03, *p* = 0.32
Average sleep duration (hours)	7.8 (0.74)	7.7 (0.41)	*t*_(13.7)_ = 0.37, *p* = 0.72

Values are reported as mean (SD) unless otherwise indicated.

### Scale playing: extracting correct scales

We recorded a total of 463 note onsets (*SD* = 53) for each pianist, hand, and recording (AM or PM). More note onset material was recorded for the left (*M* = 482, *SD* = 58 onsets) than for the right hand (*M* = 446, *SD* = 40 onsets) [*F*_(1, 19)_ = 19.76, *p* = 0.003, η^2^_G_= 0.06]. From these, we extracted the correct inward and outward scales, discarding onsets that were part of incomplete scales or scales with errors (786 keystrokes discarded, amounting to 1.9% of the recorded material). We found 15.0 (*SD* = 2.50) correctly produced scales for each participant, hand, playing direction (inward or outward) and recording. Participants' left hand playing contained more correct scales (*M* = 15.6, *SD* = 1.75) than the right hand (*M* = 14.6, *SD* = 1.28) [*F*_(1, 19)_ = 15.89, *p* < 0.001, η^2^_G_ = 0.05]. There was no difference in number of correct scales according to the inward or outward playing direction [*F*_(1, 19)_ = 1.11, *p* = 0.3]. There was a trend for there to be more correctly played scales in the PM recording (*M* = 15.7, *SD* = 2.16 scales) than in the AM recording (*M* = 14.5, *SD* = 2.30 scales) [*F*_(1, 19)_ = 3.06, *p* = 0.10]. Importantly, there was no main effect of chronotype [*F*_(1, 19)_ = 0.01, *p* = 0.9], revealing that both chronotype subgroups played an equal number of correct scales. There was an interaction between playing direction and time-point of recording [*F*_(1, 19)_ = 6.70, *p* = 0.02, η^2^_G_ = 0.002], revealing that for the PM recording there were more correct inward than correct outward scales recorded, whereas for the AM recording the opposite was true. None of the other two-way interactions was significant.

### Scale playing: unevenness

First we calculated the traditional measure of *unevenness*, defined as the standard deviation of the intervals between the onsets of subsequent keystrokes. There was no main effect of playing direction (inward vs. outward) [*F*_(1, 19)_ = 0.06, *p* = 0.4] or recording time-point [*F*_(1, 19)_ = 2.79, *p* = 0.11]. However, there was a main effect of hand [*F*_(1, 19)_ = 26.35, *p* < 0.001, η^2^_G_ = 0.10], indicating that the right hand played more evenly (*M* = 10.4, *SD* = 2.12 ms) than the left hand (*M* = 12.3, *SD* = 2.64 ms). There was no main effect of chronotype [*F*_(1, 19)_ = 0.03, *p* = 0.87]. There was a trend for an interaction between hand and direction [*F*_(1, 19)_ = 4.14, *p* = 0.06, η^2^_G_ = 0.01], indicating that for the left hand, outward scales were more uneven than inward scales, whereas for the right hand the opposite was true. None of the two-way interactions were significant [all *F*_(1, 19)_ < 2.18, *p* > 0.15], nor any of the three-way interactions [all *F*_(1, 19)_ < 1.65, *p* > 0.2] or the four-way interaction [*F*_(1, 19)_ = 1.89, *p* = 0.18].

### Scale playing: irregularity and instability

We proceeded to calculate the irregularity-instability analysis, which decomposes the variability in scale playing variability consistent timing deviations (*irregularity*) and trial-to-trial variability (*instability*) (van Vugt et al., 2012).

First, we report the results of the irregularity analysis. There was no main effect of pianist chronotype [*F*_(1, 19)_ = 0.02, *p* = 0.87] or playing direction [*F*_(1, 19)_ = 1.18, *p* = 0.29]. We found a trend for a main effect of recording time-point [*F*_(1, 19)_ = 3.31, *p* = 0.08], showing that playing tended to be slightly more regular during the PM recordings (*M* = 6.14, *SD* = 1.41 ms) than during the AM recordings (*M* = 6.54, *SD* = 1.59 ms). Similarly, a trend for a main effect of hand [*F*_(1, 19)_ = 3.71, *p* = 0.07] showed that right hand playing tended to be more regular (*M* = 6.08, *SD* = 1.53 ms) than the left hand (*M* = 6.60, *SD* = 1.54 ms). There was an interaction between hand and playing direction [*F*_(1, 19)_ = 5.81, *p* = 0.03, η^2^_G_ = 0.03], indicating that the right hand outward scales were more regular than the inward scales, whereas for the left hand the opposite was true. Importantly, there was no interaction between pianist chronotype and recording time point [*F*_(1, 19)_ = 2.89, *p* = 0.11]. None of the other two-, three- or four-way interactions was significant [all *F*_(1, 19)_ < 1.08, *p* > 0.3].

**Figure 2:**
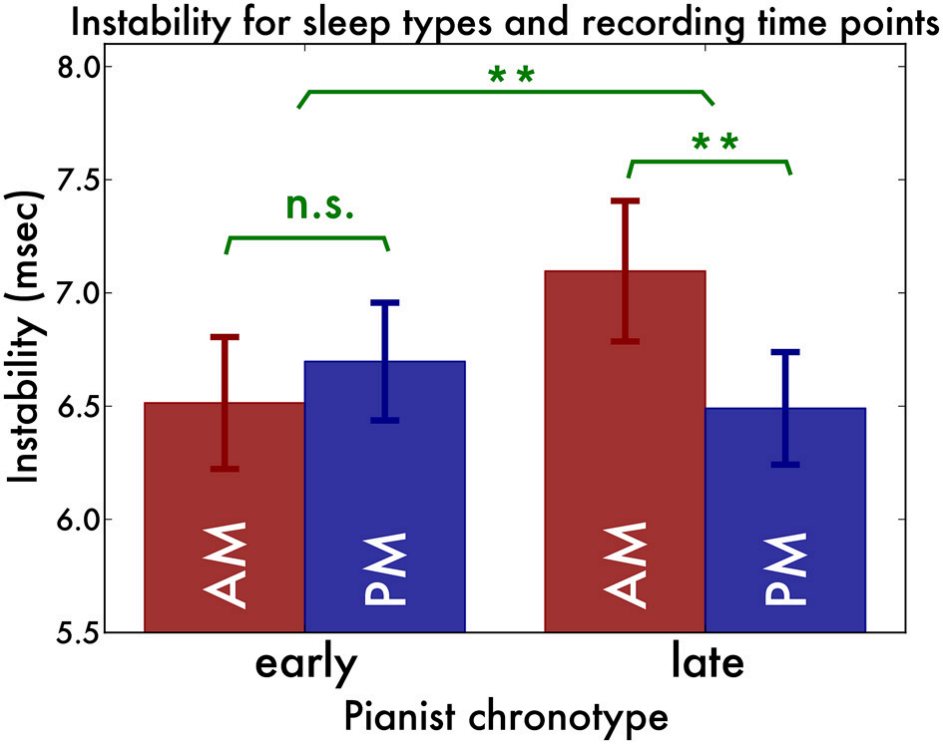
**Playing instability (trial-to-trial variability) for the early and late chronotypes and the morning (red bars) and evening (blue bars) recordings.** We found that early sleepers' playing was equally stable in both recordings, whereas late sleepers' playing was more stable in the evening than in the morning. ^*^p < 0.05, ^**^p < 0.01.

The instability revealed a different picture. A main effect of hand [*F*_(1, 19)_ = 22.47, *p* < 0.001, η^2^_G_ = 0.14] revealed that the left hand played more unstable (*M* = 7.16, *SD* = 1.11 ms) than the right hand (*M* = 6.25, *SD* = 0.76 ms). A main effect of direction [*F*_(1, 19)_ = 21.78, *p* < 0.001, η^2^_G_ = 0.03] indicated that timing in outward scales was more stable (*M* = 6.50, *SD* = 0.79 ms) than inward scales (*M* = 6.91, *SD* = 0.95 ms). There was no main effect of pianist chronotype [*F*_(1, 19)_ = 0.25, *p* = 0.62]. There was an interaction between scale direction and recording time-point [*F*_(1, 19)_ = 4.52, *p* = 0.05, η^2^_G_ = 0.01], which indicated that inward scales were more stable at the PM recording, whereas outward scales were equally stable during both recordings. Now, crucially, we found an interaction effect between pianist chronotype and recording time-point [*F*_(1, 19)_ = 10.20, *p* = 0.004, η^2^_G_ = 0.03], which indicated that pianists of the late chronotype played more stably in the PM recording than in the AM recording [*F*_(1, 10)_ = 15.92, *p* = 0.003, η^2^_G_ = 0.06] whereas pianists of the early chronotype played equally stable in both recordings [*F*_(1, 9)_ = 0.85, *p* = 0.38] (Figure 2).

In order to assess how sensitive this result is to the intermediate chronotypes (those close to the median split), we performed a control analysis. In this analysis, we included only the 7 earliest chronotype pianists and the 7 latest chronotype pianists. With instability as a dependent variable, the same interaction between chronotype and recording time point was found [*F*_(2, 12)_ = 7.17, *p* = 0.02, η^2^_G_= 0.05]. Again, no such interaction was present with irregularity as dependent variable [*F*_(2, 12)_ = 2.21, *p* = 0.16].

**Figure 3:**
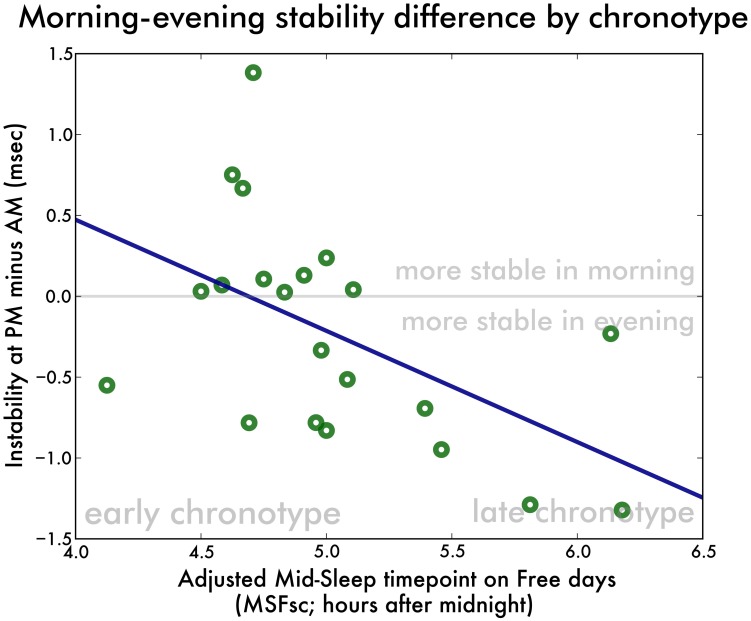
**Difference in playing instability between the morning and evening recordings as a function of pianist chronotype.** We have collapsed the two hands and two directions to yield a single data point for each participant. Positive instability differences denote more stable playing in the morning. A negative correlation is observed, indicating that the later the chronotype, the more stable the scale playing is in the evening relative to the morning.

In order to eliminate problems due to the small sample size of our two groups, we performed the following analysis of co-variance (ANCOVA) on our entire participant pool. First, we calculated the difference in irregularity and instability between the morning and evening recording for each pianist, hand and direction. We then performed a ANCOVA with the irregularity difference as outcome variable and the pianist chronotype as independent variable, allowing for variable slope and intercept according to hand and direction. The model did not reach significance [*F*_(5, 78)_ = 1.69, *p* = 0.15], further supporting the notion that irregularity differences between morning and evening recordings are not systematically influenced by pianist chronotype. We then performed the same regression with instability as a dependent variable and found a significant model [*F*_(5, 78)_ = 2.59, *p* = 0.03]. The mid-sleep time point on free days (MSFsc) was a significant predictor of the morning-evening instability difference [*t* = −2.24, *p* = 0.03] (Figure 3). The slope and intercept of the regression line were not different according to hand [both *t* < 0.13, *p* > 0.89] or playing direction [both *t* < 0.91, *p* > 37]. This regression corresponds to a negative correlation [*r*_(19)_ = −0.52, *p* = 0.01]. The same analysis with unevenness as outcome variable did not yield a significant model [*F*_(5, 78)_ = 0.78, *p* = 0.56] nor a correlation [*r*_(19)_ = −0.22, *p* = 0.33].

### Scale playing: keystroke velocity

Thus far, we have restricted our attention to timing precision of the note onsets. Are other musical parameters affected? For each scale run, we took the average keystroke velocity and then took the median of these for each participant and condition. An ANOVA with this keystroke velocity as dependent variable revealed no main effect of hand or chronotype [both *F*_(1, 19)_ < 3.00, *p* > 0.14]. However, there was a main effect of scale direction [*F*_(1, 19)_ = 5.69, *p* < 0.03, η^2^_G_ = 0.01], revealing that outward scales were played louder (*M* = 85.4, *SD* = 4.1 MIDI units) than inward scales (*M* = 84.6, *SD* = 4.4 MIDI units). Furthermore, there was a main effect of recording [*F*_(1, 19)_ = 10.21, *p* = 0.005, η^2^_G_ = 0.03], which indicated that keystroke velocity during AM recordings was slower (*M* = 84.2, *SD* = 4.4 MIDI units) than using the PM recordings (*M* = 85.8, *SD* = 4.3 MIDI units). There was a trend for an interaction between pianist chronotype and recording [*F*_(1, 19)_ = 4.22, *p* = 0.05, η^2^_G_ = 0.01]. This revealed that the keystroke velocity increase between the AM and PM recordings was negligible for early chronotype pianists (*M* = 0.5, *SD* = 2.5 MIDI units) but considerable for late chronotype pianists (*M* = 2.5, *SD* = 2.1 MIDI units).

We continued to analyse the keystroke velocity unevenness (the median of the SD of the keystrokes in each scale run). We found no main effect of chronotype [*F*_(1, 19)_ = 1.26, *p* = 0.27] but there was a main effect of hand [*F*_(1, 19)_ = 5.14, *p* = 0.04, η^2^_G_ = 0.02], revealing that keystroke velocities were more even in the right (*M* = 5.8, *SD* = 1.5 MIDI units) than in the left hand (*M* = 6.2, *SD* = 1.3 MIDI units). There were no other main effects [both *F*_(1, 19)_ < 0.87]. There was an interaction effect of hand and direction [*F*_(1, 19)_ = 24.69, *p* < 0.001, η^2^_G_ = 0.07], revealing that for the left hand the keystroke velocities of the outward scales were more even than the inward scales, whereas for the right hand the opposite was true. Crucially, there was an interaction between pianist chronotype and recording time-point [*F*_(1, 19)_ = 5.67, *p* = 0.03, η^2^_G_ = 0.02], revealing that keystroke velocities of pianists of the early chronotype were more even in the AM recordings than in the PM recordings, and vice versa for the late chronotype pianists. There were no other interactions [all *F*_(1, 19)_ < 1.83, *p* > 0.19]. We emphasize that these results in keystroke velocity unevenness should be interpreted with caution, because of the differences in baseline velocity between the groups, as well as the interaction with chronotype mentioned above.

## Discussion

We investigated the influence of chronotype on fine motor performance, taking playing of musical scales in pianists as an example. Piano students were recorded playing scales in the morning and evening. The participant pool was divided into early and late sleepers in a way that no differences in musical training, age, or gender between the groups occurred. These two groups were shown to be comparable in overall temporal precision in scale playing and also revealed no differences the temporal unevenness of scale playing between the morning and evening recording sessions.

However, the unevenness metric assesses overall variability of scale timing without taking into account that part of the variability is consistent between trials. Indeed, it has been shown that some of the notes are consistently late or early (van Vugt et al., 2012) in such a way that a highly individual temporal deviation pattern appears. This trace is mostly due to differential biomechanical properties of the motor system of individual pianists (van Vugt et al., 2013). Therefore, we expected that this trace would not be susceptible to circadian fluctuations, since basic properties of the motor system such as muscle- and joint materials remain the same throughout the day. This prediction was largely supported, as at most a slight tendency was found for circadian variation in irregularity. The residual variability, that is, the variability that diverged from this individual trace (instability), revealed a different picture. Although there was no overall difference in timing stability between the pianists, an interaction was found between the recording time and the pianists' chronotype. This revealed that late sleepers' timing was more stable in the evening than in the morning, whereas early sleepers exhibited no difference between the two recordings. Our result can therefore be added to a growing list of aspects of human performance that are subject to circadian fluctuations (Kleitman et al., 1938; Atkinson et al., 1993; Coldwells et al., 1994; Gauthier et al., 1996; Monk and Kupfer, 2000; Dosseville et al., 2002; Van Eekelen and Kerkhof, 2003). In our context, it is interesting to notice that even long-term, massed practice of these expert musicians has not been able to wash out circadian fluctuations in performance. As such, we suggest that such fluctuations are much more deeply embedded in the human motor system than might otherwise be assumed.

The question that remains open, is why the early chronotype group did not show a difference in instability between the recordings (as the late chronotype did). On the other hand, the regression analysis on both groups combined showed a significant correlation between chronotype and morning-evening instability difference. An explanation might be that our participant pool did not include sufficiently early chronotypes. Indeed, the early chronotype mid-sleep time point (MSFsc) was not early compared to a reference population reported by Roenneberg et al. (2004). Therefore, a future study could include earlier chronotypes and might reveal the recording effect not found in this study.

A secondary thread of analyses of keystroke velocities revealed that in the evening, keystroke velocities were higher than in the morning, and there was a trend for this inequality to be different between the two chronotypes. This result may be interpreted in analogy with previous findings on physical force that underlie a circadian fluctuation with a minimum in the morning and a maximum in the afternoon or early evening. Left hand maximal grip strength (Atkinson et al., 1993) was, for example, significantly higher in the evening than in the morning. A similar finding was reported for left elbow flexor torque (Gauthier et al., 1996). Although these results were reported for completely different settings it is possible that fluctuations of physical force influence piano playing: pianists may tend to exert higher forces while playing the piano as soon as the maximum available forces are higher—with the result of higher key velocities in the evening. Finally, keystroke velocity analyses revealed that early chronotypes were more even in the morning and late chronotypes more even in the evening. This finding, although in line with our hypothesis of a chronotype-specific optimal playing time in the daily cycle, should be interpreted with caution. The reason is that the baseline velocities were different between the two measurements and differentially so for the two chronotypes.

It is important to realize that our result is based on the mid-sleep time-point on free days adjusted for individual average sleep need accumulated on work days (MSFsc). The measurements themselves, however, took place on work days. In this way, we have eliminated possible influences of larger variations in sleeping behavior on free days (Roenneberg et al., 2003b). However, since many participants reported a number of 7 work days per week, the aforementioned adjustment was relevant in a minority of participants. As a consequence, the influence of the recordings being carried out on work days may be limited in our study sample. A limitation of the present study is that we did not collect data on the participants' sleep quantity and quality of night before.

On the level of the scale playing analysis methodology, we found that the left hand played the scales less evenly, and in particular with greater instability. The amount of discarded scale material (due to errors) was not different between the two hands. Our result replicates the previous finding that left hand playing was shown to be less evenly in a scale playing task, both for right- and left-handed pianists (Kopiez et al., 2011).

How salient are the differences we present here? We feel that the present result, although statistically reliable, is subtle. Timing differences were in the order of milliseconds. Previous research showed that even expert musicians are insensitive to timing irregularities below approximately 10 ms unevenness (van Vugt et al., 2013), suggesting that concert audience's appreciation is not likely to be much influenced by the performer's chronotype. However, the possibility remains that when the pianists' capacities are taken to the limit, such as in playing a challenging piano concerto over sustained (multi-hour) periods of time, these circadian fluctuations become perceptible and a determining factor in the appreciation of the performance.

A future study might aim at replicating our result but with a larger range of chronotypes. Our study has not specifically recruited extreme chronotypes but instead opted for a representative sample of the music student population. The mid-sleep time point (MSFsc) values of the students in our sample were approximately within ±1 SD of the range of the mean MSFsc values yielded in a large population for this age group (Roenneberg et al., 2004) (see Figure FA1). Our prediction is that more extreme chronotypes will show a greater difference in performance between morning and evening recordings. Additional questions for future research are whether the size of the instability difference between morning and evening recordings is different for the early and late sleepers and whether the deficit in performance skills due to circadian fluctuation may be reduced through a shift of the sleep phase prior to a performance at a chronobiologically unfavorable time of the day.

### Conflict of interest statement

The authors declare that the research was conducted in the absence of any commercial or financial relationships that could be construed as a potential conflict of interest.

## Figures and Tables

**Figure A1:**
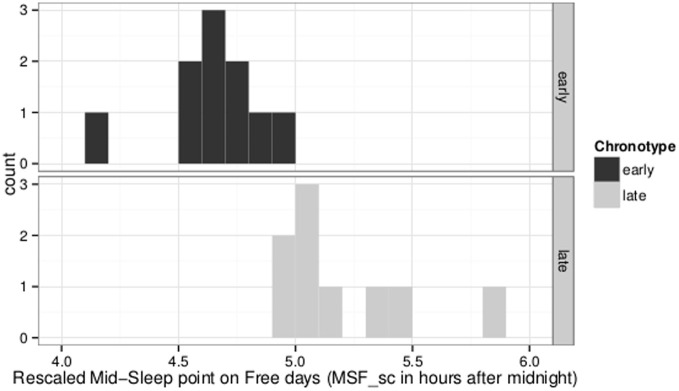
**Distribution of the adjusted mid-sleep time point on free days (MSFsc) for the early and late chronotype groups**.

## Appendix

### Questionnaire

Below are the excerpts from the questionnaire that we used to establish participant's mid-practice time and accumulated practice time. The questions are translated from German.

**Table:**

Surname:	First name:
Date of birth:	Gender (m/f):
Phone	E-mail:
Study path:	Total number of semesters:
Today's date:	

(1) How old were you when you started to play the piano?

______________ years.

(2) Please indicate the amount of your average daily practice time in hours for the respective age segment.

**Table:**

Age (years)	0–10	11–15	16–20	21–25	26–30	31–35
Hours						

(3) Please indicate the amount of your current average practice time in hours for the respective times of the day.

**Table:**

Time of the day	0–3 a.m.	3–6 a.m.	6–9 a.m.	9–12 a.m.	12–15 p.m.	15–18 p.m.	18–21 p.m.	21–24 p.m.
Hours (maximum: 3 h per cell)								

## References

[B1] Aschoff J. (1955). Der tagesgang der Körpertemperatur beim Menschen. J. Mol. Med. 33, 545–551 10.1007/BF01473763 14392884

[B2] Atkinson G. Coldwells A. Reilly T. Waterhouse J. (1993). A comparison of circadian rhythms in work performance between physically active and inactive subjects. Ergonomics 36, 273–281 10.1080/00140139308967882 8440222

[B3] Bakeman R. (2005). Recommended effect size statistics for repeated measures designs. Behav. Res. Methods 37, 379–384 10.3758/BF03192707 16405133

[B4] Benoit O. Foret J. Merle B. Bouard G. (1981). Diurnal rhythm of axillary temperature in long and short sleepers: effects of sleep deprivation and sleep displacement. Sleep 4, 359–365 7198285 10.1093/sleep/4.4.359

[B5] Coldwells A. Atkinson G. Reilly T. (1994). Sources of variation in back and leg dynamometry. Ergonomics 37, 79–86 10.1080/00140139408963625 8112285

[B6] Dosseville F. Moussay S. Larue J. Gauthier A. Davenne D. (2002). Physical exercise and time of day: influences on spontaneous motor tempo. Percept. Mot. Skills 95, 965–972 12509204 10.1177/003151250209500301

[B7] Gauthier A. Davenne D. Martin A. Cometti G. Hoecke J. V. (1996). Diurnal rhythm of the muscular performance of elbow flexors during isometric contractions. Chronobiol. Int. 13, 135–146 10.3109/07420529609037077 8877122

[B8] Hasher L. Zacks R. T. Rahhal T. A. (1999). Timing, instructions, and inhibitory control: some missing factors in the age and memory debate. Gerontology 45, 355–357 10.1159/000022121 10559658

[B9] Horne J. A. Ostberg O. (1976). A self-assessment questionnaire to determine morningness-eveningness in human circadian rhythms. Int. J. Chronobiol. 4, 97–110 1027738

[B10] Intons-Peterson M. J. Rocchi P. West T. McLellan K. Hackney A. (1999). Age, testing at preferred or nonpreferred times (testing optimality), and false memory. J. Exp. Psychol. Learn. Mem. Cogn. 25, 23 10.1037/0278-7393.25.1.23 9949706

[B11] Jabusch H.-C. Vauth H. Altenmüller E. (2004). Quantification of focal dystonia in pianists using scale analysis. Mov. Disord. 19, 171–180 10.1002/mds.10671 14978672

[B12] Jasper I. Roenneberg T. Häußler A. Zierdt A. Marquardt C. Hermsdörfer J. (2010). Circadian rhythm in force tracking and in dual task costs. Chronobiol. Int. 27, 653–673 10.3109/07420521003663793 20524807

[B13] Kantermann T. Forstner S. Halle M. Schlangen L. Roenneberg T. Schmidt-Trucksäss A. (2012). The stimulating effect of bright light on physical performance depends on internal time. PLoS ONE 7:e40655 10.1371/journal.pone.0040655 22808224 PMC3394763

[B14] Kleitman N. Titelbaum S. Feiveson P. (1938). The effect of body temperature on reaction time. Am. J. Physiol. Legacy Content 121, 495–501

[B15] Kopiez R. Jabusch H.-C. Galley N. Homann J.-C. Lehmann A. C. Altenmuller E. (2011). No disadvantage for left-handed musicians: the relationship between handedness, perceived constraints and performance-related skills in string players and pianists. Psychol. Music 40, 357–384 10.1177/0305735610394708

[B16] Matchock R. L. Mordkoff J. T. (2009). Chronotype and time-of-day influences on the alerting, orienting, and executive components of attention. Exp. Brain Res. 192, 189–198 10.1007/s00221-008-1567-6 18810396

[B17] May C. P. Hasher L. Stoltzfus E. R. (1993). Optimal time of day and the magnitude of age differences in memory. Psychol. Sci. 4, 326–330 10.1111/j.1467-9280.1993.tb00573.x

[B18] Merrow M. Spoelstra K. Roenneberg T. (2005). The circadian cycle: daily rhythms from behaviour to genes. EMBO Rep. 6, 930–935 10.1038/sj.embor.7400541 16222241 PMC1369194

[B19] Monk T. H. Kupfer D. J. (2000). Circadian rhythms in healthy aging-effects downstream from the pacemaker. Chronobiol. Int. 17, 355–368 10.1081/CBI-100101051 10841210

[B20] Münte T. F. Altenmüller E. Jäncke L. (2002). The musician's brain as a model of neuroplasticity. Nat. Rev. Neurosci. 3, 473–478 10.1038/nrn843 12042882

[B21] Peres I. Vetter C. Blautzik J. Reiser M. Pöppel E. Meindl T. (2011). Chronotype predicts activity patterns in the neural underpinnings of the motor system during the day. Chronobiol. Int. 28, 883–889 10.3109/07420528.2011.619084 22080733

[B22] Petros T. V. Beckwith B. E. Anderson M. (1990). Individual differences in the effects of time of day and passage difficulty on prose memory in adults. Br. J. Psychol. 81, 63–72 10.1111/j.2044-8295.1990.tb02346.x

[B23] Reilly T. Atkinson G. Edwards B. Waterhouse J. Farrelly K. Fairhurst E. (2007). Diurnal variation in temperature, mental and physical performance, and tasks specifically related to football (soccer). Chronobiol. Int. 24, 507–519 10.1080/07420520701420709 17612948

[B24] Roenneberg T. Daan S. Merrow M. (2003a). The art of entrainment. J. Biol. Rhythms 18, 183–194 10.1177/0748730403018003001 12828276

[B25] Roenneberg T. Wirz-Justice A. Merrow M. (2003b). Life between clocks: daily temporal patterns of human chronotypes. J. Biol. Rhythms 18, 80–90 10.1177/0748730402239679 12568247

[B26] Roenneberg T. Kuehnle T. Pramstaller P. P. Ricken J. Havel M. Guth A. (2004). A marker for the end of adolescence. Curr. Biol. 14, R1038–R1039 10.1016/j.cub.2004.11.039 15620633

[B27] Roenneberg T. Merrow M. (2003). The network of time: understanding the molecular circadian system. Curr. Biol. 13, R198–R207 10.1016/S0960-9822(03)00124-6 12620213

[B28] Tamm A. S. Lagerquist O. Ley A. L. Collins D. F. (2009). Chronotype influences diurnal variations in the excitability of the human motor cortex and the ability to generate torque during a maximum voluntary contraction. J. Biol. Rhythms 24, 211–224 10.1177/0748730409334135 19465698

[B29] Van Eekelen A. P. J. Kerkhof G. A. (2003). No interference of task complexity with circadian rhythmicity in a constant routine protocol. Ergonomics 46, 1578–1593 10.1080/0014013031000121598 14668176

[B30] van Vugt F. T. Jabusch H.-C. Altenmüller E. (2012). Fingers phrase music differently: trial-to-trial variability in piano scale playing and auditory perception reveal motor chunking. Front. Psychol. 3:495 10.3389/fpsyg.2012.00495 23181040 PMC3499913

[B31] van Vugt F. T. Jabusch H.-C. Altenmüller E. (2013). Individuality that is unheard of: systematic temporal deviations in scale playing leave an inaudible pianistic fingerprint. Front. Psychol. 4:134 10.3389/fpsyg.2013.00134 23519688 PMC3604639

[B32] Wagner C. (1971). The influence of the tempo of playing on the rhythmic structure studied at pianist's playing scales, in Medicine and Sport Biomechanics II, eds Vredenbregt J. Wartenweiler J. (Basel: Karger), 129–132

[B33] West R. Murphy K. J. Armilio M. L. Craik F. I. M. Stuss D. T. (2002). Effects of time of day on age differences in working memory. J. Gerontol. B Psychol. Sci. Soc. Sci. 57, P3–P10 10.1093/geronb/57.1.P3 11773218

[B34] Wisser H. Breuer H. (1981). Circadian changes of clinical chemical and endocrinological parameters. J. Clin. Chem. Clin. Biochem. 19, 323–337 7024460

